# Attributable Fraction of Influenza Virus Detection to Mild and Severe Respiratory Illnesses in HIV-Infected and HIV-Uninfected Patients, South Africa, 2012–2016

**DOI:** 10.3201/eid2307.161959

**Published:** 2017-07

**Authors:** Stefano Tempia, Sibongile Walaza, Jocelyn Moyes, Adam L. Cohen, Claire von Mollendorf, Meredith L. McMorrow, Florette K. Treurnicht, Marietjie Venter, Marthi Pretorius, Orienka Hellferscee, Nicole Wolter, Anne von Gottberg, Athermon Nguweneza, Johanna M. McAnerney, Halima Dawood, Ebrahim Variava, Shabir A. Madhi, Cheryl Cohen

**Affiliations:** National Health Laboratory Service, Johannesburg, South Africa (S. Tempia, S. Walaza, J. Moyes, C. von Mollendorf, F.K. Treurnicht, M. Pretorius, O. Hellferscee, N. Wolter, A. von Gottberg, A. Nguweneza, J.M. McAnerney, S.A. Madhi, C. Cohen);; US Centers for Disease Control and Prevention, Atlanta, Georgia, USA (S. Tempia, A.L. Cohen, M.L. McMorrow);; US Centers for Disease Control and Prevention, Pretoria, South Africa (S. Tempia, A.L. Cohen, M.L. McMorrow);; University of the Witwatersrand, Johannesburg (S. Walaza, J. Moyes, C. von Mollendorf, O. Hellferscee, N. Wolter, A. von Gottberg, E. Variava, S.A. Madhi, C. Cohen);; World Health Organization, Geneva, Switzerland (A.L. Cohen); University of Pretoria, Pretoria (M. Venter, M. Pretorius);; Pietermaritzburg Metropolitan Hospital, Pietermaritzburg, South Africa (H. Dawood); University of KwaZulu-Natal, Pietermaritzburg (H. Dawood);; Klerksdorp-Tshepong Hospital Complex, Klerksdorp, South Africa (E. Variava)

**Keywords:** influenza, attributable fraction, respiratory illness, viruses, HIV, HIV-infected patients, HIV-uninfected patients, HIV/AIDS and other retroviruses, South Africa

## Abstract

The attributable fraction (AF) of influenza virus detection to illness has not been described for patients in different age groups or with different HIV infection statuses. We compared the age group–specific prevalence of influenza virus infection among patients with influenza-like illness (ILI) or severe acute or chronic respiratory illness (SARI and SCRI, respectively) with that among controls, stratified by HIV serostatus. The overall AF for influenza virus detection to illness was 92.6% for ILI, 87.4% for SARI, and 86.2% for SCRI. Among HIV-uninfected patients, the AF for all syndromes was highest among persons <1 and >65 years of age and lowest among persons 25–44 years of age; this trend was not observed among HIV-infected patients. Overall, influenza viruses when detected in patients with ILI, SARI, or SCRI are likely attributable to illness. This finding is particularly likely among children and the elderly irrespective of HIV serostatus and among HIV-infected persons irrespective of age.

Influenza virus infections cause substantial illness and death worldwide, in particular among persons <5 and >65 years of age and among persons with underlying medical conditions, including HIV infection (*1*–*6*). In recent years, advances in the development of sensitive PCR assays have considerably expanded the ability of laboratories to detect pathogens, especially viral agents. Such assays have been used for clinical diagnostics, surveillance, and assessment of disease burden.

Nonetheless, establishing a clinical association between pathogen detection and illness remains challenging. Contradicting results have been reported about the association of influenza virus detection with illness (*7*–*11*). These differences may be due to different study designs, including the selection of controls and geographic distribution of cases. Furthermore, this association is poorly understood among different age groups and HIV-infected persons, even though children <5 years of age, the elderly, and HIV-infected persons are recommended groups for influenza immunization (*12*).

Improving the understanding of the association between influenza virus detection and disease could provide insight into the vaccine-preventable fraction of illness overall and among groups recommended for influenza immunization. In addition, a better understanding of this association could assist with the interpretation of surveillance data and improve disease burden estimates.

We assessed the attributable fraction (AF) of influenza virus detection to illness (an estimate of the proportion of patients positive for influenza virus who have symptomatic illness resulting from that virus; hereafter referred as influenza virus AF) among HIV-infected and HIV-uninfected persons in different age groups who sought medical care for influenza-like illness (ILI); severe acute respiratory illness (SARI) [i.e., syndromes recommended by the World Health Organization (WHO) for global influenza surveillance (*13*)]; or severe chronic respiratory illness (SCRI) at selected sentinel sites in South Africa during 2012–2016. In addition, we separately assessed the AF of influenza virus types and subtypes.

## Methods

### Surveillance among Hospitalized Persons

During May 2012–April 2016, we conducted prospective hospital-based surveillance for SARI and SCRI at 3 public hospitals in 2 South Africa provinces: the Edendale Hospital in a periurban area of KwaZulu-Natal Province and the Klerksdorp and Tshepong Hospitals (the Klerksdorp-Tshepong Hospital Complex) in a periurban area of North West Province. We defined a case of SARI as illness in a hospitalized patient who had symptom onset within 10 days before admission and who met age-specific clinical inclusion criteria. Cases in infants 2 days to <3 months of age included any hospitalized patients in this age group with a diagnosis of suspected sepsis or physician-diagnosed acute lower respiratory tract infection, irrespective of signs and symptoms. Cases in children 3 months to <5 years of age included any hospitalized patients in this age group with physician-diagnosed acute lower respiratory tract infection (e.g., bronchitis, bronchiolitis, and pneumonia) or pleural effusion. Cases in children >5 years of age included any hospitalized patients in this age group with manifestation of acute lower respiratory tract infection with recorded temperature >38°C or history of fever and cough. A case of SCRI was defined as illness in a hospitalized person who had symptom onset >10 days before admission and who met the age-specific clinical inclusion criteria described above.

### Surveillance among Persons at Outpatient Clinics

During May 2012–April 2016, we also conducted prospective surveillance for persons with ILI and for asymptomatic persons (controls) at 2 outpatient clinics located in the catchment areas of the hospitals where we conducted SARI and SCRI surveillance: the Edendale Gateway Clinic in KwaZulu-Natal Province and the Jouberton Clinic in North West Province. We defined a case of ILI as illness in an outpatient of any age who sought medical care at 1 of the sentinel clinics and had a recorded temperature >38°C or a history of fever and cough of <10 days’ duration. We defined controls as persons of any age who sought medical care at 1 of the sentinel clinics for reasons other than fever or respiratory or gastrointestinal symptoms during the 14 days preceding the visit. Most controls visited the clinics for dental procedures, family planning, well baby visits, voluntary HIV counseling and testing, or acute care for nonfebrile illnesses. We aimed to enroll 1 HIV-infected and 1 HIV-uninfected control every week in each clinic within each of the following age categories: <1 year, 1–4 years, 5–24 years, 25–44 years, 45–64 years, and >65 years.

### Study Procedures

The procedures for these surveillance programs have been previously described (*4*,*7*,*14*,*15*). In brief, study staff completed case report forms for all enrolled controls and for patients with ILI, SARI, or SCRI. Referral to hospital was recorded for all enrolled patients with ILI, and patients with ILI who were referred to a hospital were excluded from the analysis.

### Laboratory Procedures

We collected respiratory specimens from all persons enrolled in the study (i.e., controls and patients with ILI, SARI, or SCRI); specimens consisted of nasopharyngeal aspirates for children <5 years of age and nasopharyngeal and oropharyngeal swabs for persons >5 years of age. When collected, the specimens were placed in universal transport medium, stored at 4°C–8°C, and transported within 72 hours of collection to the National Institute for Communicable Diseases of the National Health Laboratory Service in Johannesburg, South Africa, for testing. We used a multiplex real-time reverse transcription PCR assay (*16*) to test the specimens for 10 respiratory viruses: influenza A and B viruses; parainfluenza virus types 1, 2, and 3; respiratory syncytial virus; adenovirus; rhinovirus; human metapneumovirus; and enterovirus. We further subtyped influenza A–positive specimens (*17*).

### Determination of HIV Status

We obtained HIV status for study participants from 2 sources: patient clinical records, when available; and, for consenting study participants, by testing dried blood spots at the National Institute for Communicable Diseases. We used an HIV ELISA to test samples from all patients >18 months of age, and we used PCR to test samples from children <18 months of age if the ELISA was reactive.

### Statistical Analyses

We used unconditional logistic regression or exact logistic regression to estimate the AF of influenza-associated hospitalizations and outpatient consultations by comparing the influenza virus detection rate among patients with ILI, SARI, or SCRI with that among controls. The attributable fraction was estimated from the odds ratio (OR) obtained from the regression models as follows:

We subsequently estimated the influenza virus detection rate associated with illness among patients with ILI, SARI, or SCRI (*Infl_DetectRateIll_*) from the observed detection rate (*Infl_DetectRateObs_*) as follows:

*Infl_DetectRateIll_ = Infl_DetectRateObs_* × *AF*

We implemented this analysis overall and by HIV serostatus within the following age categories: <1 year, 1–4 years, 5–24 years, 25–44 years, 45–64 years, >65 years, <5 years, and >5 years of age. All estimates were adjusted for co-infections with the other respiratory viruses investigated in this study and underlying medical conditions known as risk factors for influenza-associated severe illness (*18*). HIV infection and age (i.e., <1 year, 1–4 years, 5–24 years, 25–44 years, 45–64 years, >65 years of age) were also included as covariates in the non-HIV–stratified overall model and in the models implemented among children <5 year of age (i.e., <1 and 1–4 years of age) and persons >5 years of age (i.e., 5–24, 25–44, 45–64 and ≥65 years of age). We also evaluated the AF of influenza virus types and subtypes overall and by HIV serostatus, using the same approach. For this analysis we also adjusted for co-circulating influenza virus types and subtypes.

In addition, we implemented the described analysis after reclassifying the SARI and SCRI cases by using a duration of symptoms cutoff of 7 days. This classification was in accordance with practices previously recommended by WHO for global influenza surveillance (*13*). We implemented this analysis to assess the influenza virus AF among SARI cases as previously defined in other Africa studies (*19*), including South Africa (*4*,*14*,*15*).

To assess trends in the magnitude of the influenza virus AF among HIV-infected and HIV-uninfected persons across age groups, we used linear regression with the inclusion of first-order (model 1, a linear model) or first-order and second-order (model 2, a quadratic model) polynomial terms for age categories treated as a continuous numerical variable. We used STATA version 14.1 (StataCorp LLP, College Station, Texas, USA) to implement the statistical analyses.

### Ethics Approval

The protocol for patients with SARI and SCRI was approved by the University of the Witwatersrand Human Research Ethics Committee (HREC; protocol no. M081042) and the University of KwaZulu-Natal Human Biomedical Research Ethics Committee (BREC; protocol no. BF157/08). The protocol for controls and patients with ILI was approved by HREC (protocol no. M120133) and BREC (protocol no. BF080/12). The surveillance was deemed nonresearch by the Centers for Disease Control and Prevention.

## Results

### Study Population

During May 2012–April 2016, we enrolled 14,431 persons, of whom 13,335 (92.4%) had known age and available influenza and HIV results and were thus included for further analyses. Of these 13,335 persons, 2,504 (18.8%) were controls; 4,797 (36.0%) had ILI; 3,755 (28.2%) had SARI; and 2,279 (17.1%) had SCRI.

Children <5 years of age accounted for 34.9% (875/2,504) of controls; 32.6% (1,565/4,797) of patients with ILI; 53.1% (1,995/3,755) of patients with SARI; and 6.7% (153/2,279) of patients with SCRI. Overall, the HIV prevalence was 41.0% (1,026/2,504) among controls (owing to enrollment criteria for controls); 27.0% (1,295/4,797) among patients with ILI; 38.7% (1,452/3,755) among patients with SARI; and 73.1% (1,667/2,279) among patients with SCRI (p<0.001). 

The HIV prevalence among patients with ILI, SARI, or SCRI was lowest among infants <1 year of age: 2.0% (11/554) among those with ILI; 9.1% (114/1,250) among those with SARI; and 20.2% (20/99) among those with SCRI. The HIV prevalence among these patients was highest among persons 25–44 years of age: 60.4% (825/1,366) among those with ILI; 89.0% (745/837) among those with SARI; and 91.6% (1,007/1,099) among those with SCRI. The median duration of illness was 2 days among patients with ILI, 3 days among patients with SARI, and 16 days among patients with SCRI.

We detected influenza viruses in 1,082 (8.1%) of 13,335 specimens. Of these, 466 (43.1%) were influenza A(H3N2) viruses; 227 (21.0%) were influenza A(H1N1)pdm09 viruses; 20 (1.8%) were influenza A viruses that were not subtyped; and 369 (34.1%) were influenza B viruses (Technical Appendix Figure 1).

### Influenza Virus Detection Rate and AF

During the study period, we detected influenza viruses in 32 (1.3%) of the 2,504 controls; 666 (13.9%) of the 4,797 patients with ILI; 251 (6.7%) of the 3,755 patients with SARI; and 133 (5.8%) of the 2,279 patients with SCRI (p<0.001) (Table 1). Among influenza-positive patients >5 years of age with severe illness (SARI or SCRI), 46.6% (117/251) had symptoms for >10 days, compared with 12.0% (16/133) among children <5 years of age (p<0.001). Among controls, after we adjusted by age, the influenza virus detection rate was statistically significantly higher among HIV-uninfected compared with HIV-infected persons (adjusted OR 1.8; 95% CI 1.1–2.7) (for HIV-infected compared with HIV-uninfected persons, adjusted OR 0.5; 95% CI 0.1–0.9).

**Table 1:** Persons positive for influenza virus infection in a study of the attributable fraction of influenza virus detection to mild and severe respiratory illnesses, Klerksdorp and Pietermaritzburg, South Africa, May 2012–April 2016*

Category	No. positive/no. total (%)		
	Total	HIV-infected	HIV-uninfected
Asymptomatic controls			
Age, y			
<1	2/363 (0.5)	0/37 (0.0)	2/326 (0.6)
1–4	9/512 (1.8)	3/223 (1.3)	6/289 (2.1)
5–24	11/729 (1.5)	3/323 (0.9)	8/406 (2.0)
25–44	5/386 (1.3)	2/238 (0.8)	3/148 (2.0)
45–64	4/357 (1.1)	2/173 (1.2)	2/184 (1.1)
>65	1/157 (0.6)	0/32 (0.0)	1/125 (0.8)
<5	11/875 (1.3)	3/260 (1.2)	8/615 (1.3)
>5	21/1,629 (1.3)	7/766 (0.9)	14/863 (1.6)
All	32/2,504 (1.3)	10/1,026 (1.0)	22/1,478 (1.5)
Influenza virus types/subtypes			
A	21/2,504 (0.8)	6/1,026 (0.6)	15/1,478 (1.0)
A(H3N2)	6/2,504 (0.2)	2/1,026 (0.2)	4/1,478 (0.3)
A(H1N1)pdm09	12/2,504 (0.5)	3/1,026 (0.3)	9/1,478 (0.6)
B	11/2,504 (0.4)	4/1,026 (0.4)	7/1,478 (0.5)
Outpatients with influenza-like illness			
Age, y			
<1	42/554 (7.6)	4/11 (36.4)	38/543 (7.0)
1–4	158/1,011 (15.6)	5/24 (20.8)	153/987 (15.5)
5–24	214/1,319 (16.2)	24/228 (10.5)	190/1,091 (17.4)
25–44	183/1,366 (13.4)	118/825 (14.3)	65/541 (12.0)
45–64	59/451 (13.1)	31/181 (17.1)	28/270 (10.4)
>65	10/96 (10.4)	3/26 (11.5)	7/70 (10.0)
<5	200/1,565 (12.8)	9/35 (25.7)	191/1,530 (12.5)
>5	466/3,232 (14.4)	176/1,260 (14.0)	290/1,972 (14.7)
All	666/4,797 (13.9)	185/1,295 (14.3)	481/3,502 (13.7)
Influenza virus types/subtypes			
A	443/4,797 (9.2)	125/1,295 (9.7)	318/3,502 (9.1)
A(H3N2)	310/4,797 (6.5)	94/1,295 (7.3)	216/3,502 (6.2)
A(H1N1)pdm09	119/4,797 (2.5)	25/1,295 (1.9)	94/3,502 (2.7)
B	223/4,797 (4.6)	60/1,295 (4.6)	163/3,502 (4.7)
Inpatients with severe acute respiratory illness			
Age, y			
<1	54/1,250 (4.3)	7/114 (6.1)	47/1,136 (4.1)
1–4	63/754 (8.4)	11/97 (11.3)	52/648 (8.0)
5–24	22/284 (7.7)	12/150 (8.0)	10/134 (7.5)
25–44	60/837 (7.2)	55/745 (7.4)	5/92 (5.4)
45–64	38/479 (7.9)	28/312 (9.0)	10/167 (6.0)
>65	14/160 (8.7)	5/34 (14.7)	9/126 (7.1)
<5	117/1,995 (5.9)	18/211 (8.5)	99/1,784 (5.5)
>5	134/1,760 (7.6)	100/1,241 (8.1)	34/519 (6.6)
All	251/3,755 (6.7)	118/1,452 (8.1)	133/2,303 (5.8)
Influenza virus types/subtypes			
A	170/3,755 (4.5)	74/1,452 (5.1)	96/2,303 (4.2)
A(H3N2)	98/3,755 (2.6)	42/1,452 (2.9)	56/2,303 (2.4)
A(H1N1)pdm09	70/3,755 (1.9)	32/1,452 (2.2)	38/2,303 (1.7)
B	81/3,755 (2.2)	44/1,452 (3.0)	37/2,303 (1.6)
Inpatients with severe chronic respiratory illness			
Age, y			
<1	9/99 (9.1)	2/20 (10.0)	7/79 (8.9)
1–4	7/54 (13.0)	1/16 (6.3)	6/38 (15.8)
5–24	15/202 (7.4)	9/141 (6.4)	6/61 (9.8)
25–44	53/1,099 (4.8)	48/1,007 (4.8)	5/92 (5.4)
45–64	37/661 (5.6)	22/438 (5.0)	15/223 (6.7)
>65	12/164 (7.3)	3/45 (6.7)	9/119 (7.6)
<5	16/153 (10.5)	3/36 (8.3)	13/117 (11.1)
>5	117/2,126 (5.5)	82/1,631 (5.0)	35/495 (7.1)
All	133/2,279 (5.8)	85/1,667 (5.1)	48/612 (7.8)
Influenza virus types/subtypes			
A	79/2,279 (3.5)	53/1,667 (3.2)	26/612 (4.2)
A(H3N2)	52/2,279 (2.3)	34/1,667 (2.0)	18/612 (2.9)
A(H1N1)pdm09	26/2,279 (1.1)	19/1,667 (1.1)	7/612 (1.1)
B	54/2,279 (2.4)	32/1,667 (1.9)	22/612 (3.6)

*Numbers indicate positive test result for any influenza virus. Influenza A category includes viruses that were not subtyped.

The overall influenza virus AF was 92.6% (95% CI 89.3%–94.8%) among patients with ILI; 87.4% (95% CI 81.3%–91.5%) among patients with SARI; and 86.2% (95% CI 77.7%–91.5%) among patients with SCRI (Table 2). Our findings showed that the AF did not statistically significantly differ (overlapping CIs) across the syndromes evaluated or by age group or HIV serostatus.

**Table 2:** AF and AF-adjusted prevalence of influenza viruses among participants in a study of the AF of influenza virus detection to mild and severe respiratory illnesses, Klerksdorp and Pietermaritzburg, South Africa, May 2012–April 2016*

Category	Total			HIV-infected			HIV-uninfected	
	AF, % (95% CI)	AF-adjusted prev, %		AF, % (95% CI)	AF-adjusted prev, %		AF, % (95% CI)	AF-adjusted prev, %
Outpatients with influenza-like illness								
Age, y†								
<1	96.8 (85.3–99.3)	7.4		98.4 (83.0–∞)‡	35.8		94.1 (74.9–98.6)	6.6
1–4	92.8 (82.2–97.0)	14.5		94.6 (72.2–98.9)	19.7		90.3 (77.7–95.8)	14.0
5–24	91.4 (81.3–96.0)	14.8		93.5 (77.5–98.1)	9.8		89.8 (78.8–95.1)	15.6
25–44	90.8 (82.7–95.1)	12.2		95.5 (81.8–98.9)	13.7		87.7 (60.2–96.2)	10.5
45–64	93.4 (81.6–97.6)	12.2		95.1 (78.8–98.8)	16.3		91.3 (62.8–98.0)	9.5
>65	94.3 (53.2–99.3)	9.8		96.3 (21.1–∞)‡	11.1		91.8 (27.6–99.1)	9.2
<5	93.9 (87.8–97.0)	12.0		97.3 (87.8–99.4)	25.0		91.7 (82.9–96.0)	11.5
>5	92.5 (88.2–95.2)	13.3		95.4 (88.5–97.5)	13.4		89.8 (82.4–94.1)	13.2
All	92.6 (89.3–94.8)	12.9		95.6 (91.0–97.8)	13.7		90.6 (85.5–93.9)	12.4
Influenza virus types/subtypes§								
A¶	93.3 (89.5–95.7)	8.6		96.5 (91.4–98.6)	9.4		91.5 (85.7–95.0)	8.3
A(H3N2)	97.3 (93.9–98.8)	6.3		98.6 (93.9–99.7)	7.2		96.7 (91.0–98.8)	6.0
A(H1N1)pdm09	85.4 (73.1–92.0)	2.1		91.0 (64.9–97.7)	1.7		82.9 (65.7–91.5)	2.2
B	92.7 (86.5–96.0)	4.3		94.4 (82.9–98.2)	4.3		91.6 (82.0–96.1)	4.3
Inpatients with severe acute respiratory illness								
Age (in years)†								
<1	93.5 (72.8–98.5)	4.0		94.3 (11.2–∞)‡	5.8		93.2 (71.2–98.4)	3.8
1–4	87.9 (73.7–94.5)	7.5		90.2 (61.0–97.6)	10.2		86.5 (66.4–94.6)	6.9
5–24	84.3 (59.5–93.9)	6.6		90.6 (64.6–97.5)	7.2		78.6 (13.6–95.9)	5.9
25–44	83.1 (63.3–92.3)	6.4		90.4 (60.1–97.7)	6.7		75.6 (31.8–91.3)	4.1
45–64	87.3 (63.2–95.6)	6.9		90.9 (54–9-97.7)	8.2		85.2 (29.0–96.9)	5.1
>65	94.5 (57.7–99.3)	8.2		91.5 (18.6–∞)‡	13.5		91.0 (27.9–98.9)	6.5
<5	89.3 (79.3–94.5)	5.3		91.0 (65.4–97.7)	7.7		89.1 (76.8–94.9)	5.0
>5	86.0 (76.9–91.5)	6.5		91.2 (80.3–96.1)	7.4		79.3 (59.6–89.4)	5.2
All	87.4 (81.3–91.5)	5.9		91.1 (82.2–95.5)	7.4		84.5 (74.7–90.5)	4.9
Influenza virus types/subtypes§								
A¶	86.7 (78.7–91.7)	3.9		91.3 (79.2–96.3)	4.7		81.9 (68.0–89.8)	3.4
A(H3N2)	93.6 (85.1–97.2)	2.4		94.6 (76.9–98.7)	2.7		92.1 (77.6–97.2)	2.2
A(H1N1)pdm09	80.7 (63.5–89.8)	1.5		88.1 (68.7–97.4)	1.9		71.7 (39.2–86.8)	1.1
B	88.4 (77.6–94.0)	1.9		90.3 (71.5–96.7)	2.7		89.0 (74.2–95.3)	1.4
Inpatients with severe chronic respiratory illness								
Age, y†								
<1	87.6 (70.7–99.4)	8.0		89. 3(9.3–∞)‡	8.9		88.1 (72.4–99.8)	7.8
1–4	85.2 (64.8–97.3)	11.1		88.3 (7.4–99.3)	5.6		83.9 (50.9–97.5)	13.3
5–24	81.3 (54.9–92.2)	6.0		85.3 (27.4–95.7)	5.5		78.8 (31.3–93.5)	7.7
25–44	78.7 (43.8–91.9)	3.8		84.6 (35.7–96.3)	4.1		69.5 (32.7–93.0)	3.8
45–64	82.9 (51.1–94.0)	4.6		83.1 (17.6–95.6)	4.2		81.6 (31.5–96.5)	5.5
>65	88.2 (38.4–99.0)	6.4		89.7 (12.0–∞)‡	6.0		88.0 (11.7–98.7)	6.7
<5	87.1 (73.0–97.4)	9.1		88.6 (52.4–99.0)	7.4		85.1 (71.7–98.1)	9.4
>5	82.7 (70.7–89.8)	4.5		86.9 (64.8–93.2)	4.3		80.2 (60.1–90.2)	5.7
All	86.2 (77.7–91.5)	5.0		87.4 (69.9–93.7)	4.5		82.9 (74.1–92.3)	6.5
Influenza virus types/subtypes§								
A¶	82.7 (68.6–90.5)	2.9		83.3 (55.8–93.7)	2.7		81.1 (60.5–91.0)	3.5
A(H3N2)	93.1 (81.3–97.4)	2.1		94.8 (64.1–98.6)	1.9		92.7 (75.5–97.8)	2.7
A(H1N1)pdm09	64.5 (32.6–84.6)	0.7		74.2 (17.3–93.8)	0.8		52.8 (9.7–84.5)	0.6
B	91.5 (82.1–95.9)	2.2		92.4 (80.7–97.0)	1.8		88.9 (61.9–96.8)	3.2

*AF, attributable fraction; AF-adjusted prev, prevalence attributable to illness. †The overall influenza virus AF was obtained from models adjusted for any underlying medical conditions and for other respiratory viruses investigated in this study. Underlying medical conditions were asplenia, including asplenia or sickle cell anemia; chronic illness, including chronic lung, renal, liver, or cardiac disease, diabetes mellitus, and asthma; other immunocompromising conditions (excluding HIV), including organ transplant, primary immunodeficiency, immunotherapy, and malignancy; neurologic disorders; burns; obesity; malnutrition; and prematurity. Comorbidities were considered absent in cases for which the medical records stated that the patient had no underlying medical conditions or when there was no direct reference to that condition. Prematurity and malnutrition were assessed only in analyses for children <1 and <5 years of age, respectively. Age and HIV infection were also included as predictors in nonstratified models. ‡Estimated using exact logistic regression. ‡The influenza virus AF was obtained from models adjusted for age, HIV infection, any underlying medical conditions, and other respiratory viruses investigated in this study, including co-circulating influenza virus types and subtypes. ¶Includes influenza A viruses that were not subtyped.

**Figure 1:**
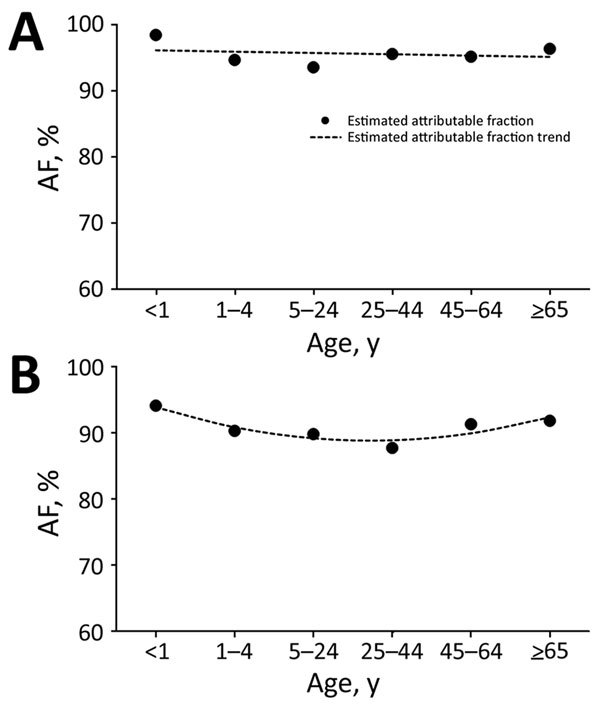
Estimated influenza virus attributable fraction (AF) and AF trends across age groups among outpatients with influenza-like illness, Klerksdorp and Pietermaritzburg, South Africa, May 2012–April 2016. A) HIV-infected patients (AF trends estimated using model 1, a linear model). B) HIV-uninfected patients (AF trends estimated using model 2, a quadratic model).

**Figure 2:**
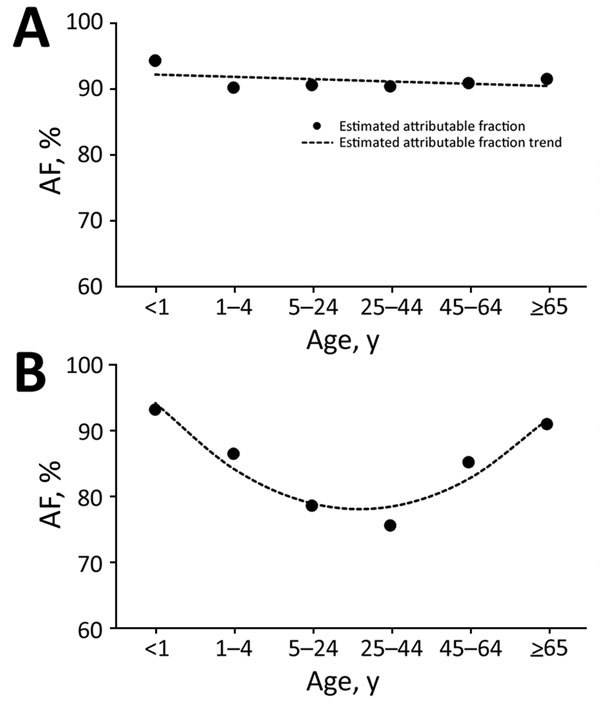
Estimated influenza virus attributable fraction (AF) and AF trends across age groups among inpatients with severe acute respiratory illness, Klerksdorp and Pietermaritzburg, South Africa, May 2012–April 2016. A) HIV-infected patients (AF trends estimated using model 1, a linear model). B) HIV-uninfected patients (AF trends estimated using model 2, a quadratic model).

**Figure 3:**
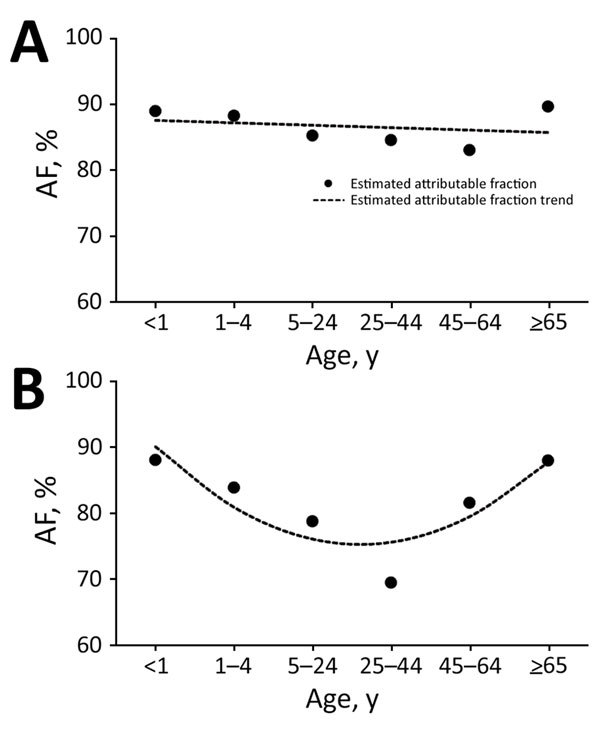
Estimated influenza virus attributable fraction (AF) and AF trends across age groups among inpatients with severe chronic respiratory illness, Klerksdorp and Pietermaritzburg, South Africa, May 2012–April 2016. A) HIV-infected patients (AF trends estimated using model 1, a linear model). B) HIV-uninfected patients (AF trends estimated using model 2, a quadratic model).

Overall, for all syndromes, the influenza virus AF was highest among persons <1 and >65 years of age and lowest among persons 25–44 years of age (Table 2; Technical Appendix Figures 2–4, panels A). The HIV-stratified analysis did not show this trend among HIV-infected persons, but it was marked among HIV-uninfected persons across syndromes (Table 2; Technical Appendix Figures 2–4, panels B, C). On linear regression analysis, we did not detect statistically significant trends of the magnitude of the influenza virus AF across age groups among HIV-infected persons for all syndromes (Figures 1–3, panels A; Technical Appendix Table 1). Conversely, we did detect a statistically significant U-shaped trend of the magnitude of the influenza virus AF across age groups among HIV-uninfected persons for all syndromes (Figures 1–3, panels B; Technical Appendix Table 1).

The AF of influenza virus types and subtypes was statistically significant across syndromes and HIV serostatus (Table 2). The influenza virus AF did not statistically significantly differ (overlapping CIs) between types and subtypes for all syndromes; however a gradient of the magnitude of the influenza virus AF was observed. The AF of influenza A(H3N2) virus was the highest, followed by that of influenza B virus and then influenza A(H1N1)pdm09 virus for all syndromes and HIV serostatus (Table 2).

Overall, the influenza virus detection rate associated with illness (AF-adjusted) was 12.9% among patients with ILI, 5.9% among those with SARI, and 5.0% among those with SCRI (Table 2). Using a duration of symptom cutoff of 7 days, we showed that the overall influenza virus detection rate among patients classified as having SARI was 6.8% and the detection rate among patients classified as having SCRI was 6.1% (Technical Appendix Table 2). We showed that the influenza virus AF was similar among patients classified as having SARI or SCRI, using a duration of symptom cutoff of 7 days (Technical Appendix Table 3) or 10 days (Table 2).

## Discussion

We provide estimates of the influenza virus AF among HIV-infected and HIV-uninfected patients of different age groups with mild or severe respiratory illness. Overall, influenza virus detection was statistically significantly associated with illness across syndromes, age groups, and HIV serostatus. Given the elevated influenza virus AF that we found in this study, the difference between the observed influenza virus detection rate and that attributable to illness was minimal. Nonetheless, among HIV-uninfected patients, the influenza virus AF was highest among persons <1 and >65 years of age and lowest among persons 25–44 years of age for all syndromes. This trend was not observed among HIV-infected patients, for whom the AF was similar across age groups for all syndromes.

A statistically significant association of influenza virus detection with illness among patients with ILI has previously been reported (*7*). A statistically significant association of influenza virus detection with illness among patients with SARI has also been reported (*7*,*10*,*11*). Nonetheless, other studies reported that respiratory syncytial virus but not influenza virus detection was associated with pneumonia in children (*8*,*9*). It should be noted that the latter studies had relatively few controls, potentially resulting in lack of power to detect statistically significant disease association for pathogens with low detection rates. A lack of studies that quantify the AF of influenza virus detection with illness among patients with SCRI hindered our ability to compare the estimates obtained in this study with those of others. In our study, the influenza virus detection rate was similar among patients with SARI (6.7%) and SCRI (5.8%). The high influenza virus AF found among patients with SCRI (86.2%) suggests that using the recommended WHO SARI case definition may underestimate the burden of influenza-associated severe illness, especially in older persons, among whom the number of patients seeking medical care for illness of longer duration is elevated. In our study, 46.6% of influenza-positive patients >5 years of age with severe illness had symptoms for >10 days, compared with 12.0% of children <5 years of age. Chronic lung diseases are a known risk factor for influenza-associated severe illness (*18*,*20*); however, illness in patients with such conditions might not be identified as SARI (because of their prolonged underlying respiratory illness), even if the patients are hospitalized after an acute infection.

The statistically significant U-shaped trends of the magnitude of the influenza virus AF found in this study among HIV-uninfected patients across age groups suggest that symptomatic illness sufficient to warrant seeking care after influenza virus infection may be more likely to develop in infants, young children, and the elderly than in young to middle-age adults. Extremes of age are known risk factors for influenza-associated severe illness (*4*–*6*,*21*), a fact that was reflected in the marked U-shaped trend of the magnitude of the influenza virus AF found in this study, especially among patients with severe illness. Statistically significant influenza virus AF has been reported among children and adults in other studies (*7*,*10*,*11*), but none of those studies were powered to assess the influenza virus AF in refined age groups.

In our study, we did not find a statistically significant trend in the magnitude of the influenza virus AF among HIV-infected persons across age groups. In addition, after adjusting by age, we found that the detection rate of influenza virus was statistically significantly lower among HIV-infected compared with HIV-uninfected controls. This finding suggests that HIV-infected persons may be more likely to develop symptomatic illness sufficient to warrant seeking care following influenza virus infection irrespective of the person’s age (*22*). Although HIV infection is a known risk factor for influenza-associated severe illness (*4*–*6*,*18*,*21*), documentation of the influenza virus AF among HIV-infected patients is lacking, hindering our ability to compare the estimates obtained in this study with those of others. 

The difference in magnitude of the influenza virus AF that we observed in our study among HIV-infected and HIV-uninfected persons across age groups may affect estimates of the relative risk for influenza-associated severe illness among persons of different age and HIV serostatus. This possibility should be investigated.

We found that the AF of influenza virus types and subtypes was statistically significant for all syndromes investigated in this study. Although we did not observe a statistically significant difference of the estimated AF between influenza virus types and subtypes, the AF of influenza A(H3N2) virus was consistently the highest and that of influenza A(H1N1)pdm09 virus was consistently the lowest across syndromes and HIV serostatus. A higher severity of influenza A(H3N2) compared with influenza A(H1N1)pdm09 and influenza B infections has been found in some studies (*23*,*24*).

Our study has limitations that warrant discussion. First, in our analysis, we did not adjust for co-infections with bacterial pathogens. Nonetheless, the synergistic effect of influenza virus with bacterial pathogens, especially *Streptococcus pneumoniae*, is well described in the literature (*25*–*27*). Specifically, studies conducted in South Africa suggest that influenza virus detection among patients with SARI is associated with increased risk for pneumococcus colonization and elevated colonization density that increases the risk for invasive pneumococcal disease and associated death, placing influenza virus infection in the pathogenesis pathway for severe respiratory illness (*15*,*28*). Second, the small sample size in some subgroups of the age- and HIV-stratified analyses hindered our ability to obtain accurate estimates of the influenza virus AF, resulting in wide CIs. Third, we did not follow up on controls after enrollment, and the development of undetected mild or severe illness for some persons cannot be excluded. Should illness have developed in some controls, the influenza virus AF in our study would have been underestimated. Fourth, we used asymptomatic persons enrolled at selected clinics as controls. Other studies have used randomly selected persons (either symptomatic or asymptomatic) in the community as controls for severe cases (*29*,*30*). We cannot dismiss that our study may have included patients who had influenza-associated mild illness but were hospitalized because of infection with other pathogens. Nonetheless, given our study design, we had to use asymptomatic controls for standard comparison across syndromes, including mild illness such as ILI. In addition, the use of controls from the community versus outpatient clinics for the estimation of AFs has not been evaluated. Fifth, we did not have data on influenza B lineages or influenza A strains in different years nor complete data on the level of immunosuppression in HIV-infected persons. This lack of data hampered our ability to estimate lineage- or strain-specific AFs and to assess the effect of different levels of immunosuppression on the influenza virus AF among HIV-infected persons.

In conclusion, influenza viruses, when detected in patients with ILI, SARI, or SCRI, are probably attributable to illness overall, especially among children and the elderly, irrespective of the HIV serostatus and among HIV-infected persons irrespective of age. Compared with the reporting of influenza virus detection rates alone, the estimated influenza virus detection rate attributable to illness reflects a more accurate description of the proportion of illness caused by influenza viruses in South Africa. Such estimates can be used to better interpret surveillance data and refine disease burden estimates so as to improve understanding of the vaccine-preventable fraction of illness overall and among groups recommended for influenza vaccination (e.g., children, the elderly, and HIV-infected persons). In addition, a differential influenza virus AF may also occur among persons with conditions associated with increased risk of influenza-associated severe illness other than age and HIV infection, and this possibility should be investigated.

Technical Appendix Detailed results for a study showing the proportion of influenza virus–positive cases attributable to mild and severe respiratory illnesses in patients with and without HIV, South Africa, 2012–2016.
