# Supplementary material for: Attributable Fraction of Influenza Virus Detection to Mild and Severe Respiratory Illnesses in HIV-Infected and HIV-Uninfected Patients, South Africa, 2012–2016
Source: Emerg Infect Dis. 2017 Jul;23(7):1124–32. doi: 10.3201/eid2307.161959 (PMC5512492; doi:10.3201/eid2307.161959)
Supplement: Technical Appendix — Detailed results for a study showing the proportion of influenza virus–positive cases attributable to mild and severe respiratory illnesses in patients with and without HIV, South Africa, 2012–2016. [file 16-1959-Techapp-s1.pdf]

Article DOI: https://dx.doi.org/10.3201/eid2307.161959

Attributable Fraction of Influenza Virus Detection to Mild or Severe Respiratory Illnesses in HIV-Infected and HIV-Uninfected Patients, South Africa, 2012–2016

Technical Appendix

Results

Patients hospitalized with severe acute (symptoms duration ≤7 days) or chronic (symptoms duration >7 days) respiratory illness

Among 6,034 inpatients with severe respiratory illness enrolled from May 2012 through April 2016 with known age and available influenza and HIV results, 2,458 (40.7%) presented with symptoms duration ≤7 days (SARI-7) and 3,576 (59.3%) presented with symptoms duration >7 days (SCRI-7).

Children aged <5 years accounted for 73.2% (1,799/2,458) and 9.7% (349/3,576) of SARI-7 and SCRI-7 cases, respectively. The HIV prevalence was 25.1% (616/2,458) among SARI-7 cases and 70.0% (2,503/3,576) among SCRI-7 cases ($p < 0.001$). Among SARI-7 and SCRI-7 cases the HIV prevalence was lowest among infants aged <1 year [SARI-7: 8.5% (98/1,154) versus SCRI-7: 18.5% (36/195)] and highest among persons aged 25–44 years [SARI-7: 89.0% (268/301) versus SCRI-7: 90.8% (1,484/1,635)].

The observed influenza virus detection rate by age group and HIV serostatus among SARI-7 and SCRI-7 cases is provided in Table 2; whereas, the influenza virus attributable fraction (AF) and the influenza virus detection rate attributable to illness (AF-adjusted) for the same population are provided in Table 3.

Technical Appendix Table 1. Trend analysis of the influenza virus attributable fraction (AF) across age groups among HIV-infected and HIV-uninfected outpatients with influenza-like illness and inpatients with severe acute (symptoms duration ≤10 d) or chronic (symptoms duration >10 d) respiratory illness, Klerksdorp and Pietermaritzburg, South Africa, May 2012–April 2016*

	Influenza-like illness			
	HIV-infected		HIV-uninfected	
Predictors	Coefficient (95% CI)	p-value	Coefficient (95% CI)	p-value
Model 1				
Age	−0.2 (−1.4 to 1.0)	0.670	−0.3 (−1.8 to 1.2)	0.613
Model 2				
Age	−3.7 (−8.1 to 0.7)	0.075	**-5.1 (–9.5 to –0.6)**	**0.037**
Age^2	0.5 (–0.1 to 1.1)	0.081	**0.7 (0.1 to 1.3)**	**0.041**
	**Severe acute respiratory illness**			
	HIV-infected		HIV-uninfected	
Predictors	Coefficient (95% CI)	p-value	Coefficient (95% CI)	p-value
Model 1				
Age	−0.3 (−1.4 to 0.7)	0.404	−0.5 (−5.5 to 4.5)	0.792
Model 2				
Age	−3.3 (−6.9 to 0.3)	0.061	**-17 (1 (–27.0 to –7.2)**	**0.012**
Age^2	0.4 (–0.1 to 0.9)	0.074	**2.4 (1.0 to 3.8)**	**0.012**
	Severe chronic respiratory illness			
	HIV-infected		HIV-uninfected	
Predictors	Coefficient (95% CI)	p-value	Coefficient (95% CI)	p-value
Model 1				
Age	−0.4 (−2.3 to 1.6)	0.627	−0.5 (−5.6 to 4.6)	0.809
Model 2				
Age	−5.7 (−13.2 to 1.9)	0.096	**-15.7 (–32.6 to –1.2)**	**0.047**
Age^2	0.7 (–0.3 to 1.8)	0.106	**2.2 (1.0 to 3.5)**	**0.038**

*Significant predictors are in bold. Model specifications: outcome variable: age-specific influenza virus attributable fraction; predictors: 1st (Model 1, a linear model) or 1st and 2nd (Model 2, a quadratic model) order polynomial terms for age categories treated as continuous numerical variable (i.e., 1: <1 y, 2: 1–4 y, 3: 5–24 y, 4: 25–44 y, 5: 45–64 y and 6: ≥65 y).

Technical Appendix Table 2. Influenza virus percentage positive among inpatients with severe acute (symptoms duration ≤7 d) or chronic (symptoms duration >7 d) respiratory illness, Klerksdorp and Pietermaritzburg, South Africa, May 2012–April 2016

	Severe acute respiratory illness (SARI-7)			Severe chronic respiratory illness (SCRI-7)		
Categories	Total % (n/N)	HIV-infected % (n/N)	HIV-uninfected % (n/N)	Total % (n/N)	HIV-infected % (n/N)	HIV-uninfected % (n/N)
Age, y*						
<1	4.2 (48/1,154)	7.1 (7/98)	3.9 (41/1,056)	7.7 (15/195)	5.6 (2/36)	8.2 (13/159)
1–4	8.2 (53/645)	10.5 (8/76)	7.9 (45/569)	11.0 (17/154)	10.8 (4/37)	11.1 (13/117)
5–24	6.2 (10/161)	4.5 (3/67)	6.4 (6/94)	8.3 (27/325)	8.0 (18/224)	8.9 (9/101)
25–44	9.6 (29/301)	10.8 (29/268)	3.0 (1/33)	5.1 (84/1,635)	5.0 (74/1,484)	6.6 (10/151)
45–64	15.4 (23/149)	17.7 (17/96)	11.3 (6/53)	5.3 (52/991)	5.1 (33/654)	5.6 (19/337)
≥65	8.3 (4/48)	9.1 (1/11)	8.1 (3/37)	7.8 (22/276)	10.3 (7/68)	7.2 (15/208)
<5	5.6 (101/1,799)	8.6 (15/174)	5.3 (86/1,625)	9.2 (32/349)	8.2 (6/73)	9.4 (26/276)
≥5	10.0 (66/659)	11.3 (50/442)	7.4 (16/217)	5.7 (185/3,227)	5.4 (132/2,430)	6.7 (53/797)
All	6.8 (167/2,458)	10.6 (65/616)	5.5 (102/1,842)	6.1 (217/3,576)	5.5 (138/2,503)	7.4 (79/1,073)
Influenza virus types/subtypes						
A†	4.7 (116/2,458)	6.3 (39/616)	4.2 (77/1,842)	3.7 (133/3,576)	3.6 (91/2,503)	3.9 (42/1,073)
A(H3N2)	3.1 (75/2,458)	4.4 (27/616)	2.6 (48/1,842)	2.1 (75/3,576)	2.0 (50/2,503)	2.3 (25/1073)
A(H1N1)pdm09	1.6 (39/2,458)	2.0 (12/616)	1.5 (27/1,842)	1.6 (57/3,576)	1.6 (41/2,503)	1.5 (16/1,073)
B	2.1 (84/2,458)	4.2 (26/616)	1.4 (25/1,842)	2.3 (84/3,576)	1.9 (47/2,503)	3.4 (37/1,073)

*Any influenza virus.
†Includes influenza A viruses that were not subtyped.

Technical Appendix Table 3. The attributable fraction (AF) and the prevalence attributable to illness (AF-adjusted prevalence) of influenza viruses among inpatients with severe acute (symptoms duration ≤7 d) or chronic (symptoms duration >7 d) respiratory illness, Klerksdorp and Pietermaritzburg, South Africa, May 2012–April 2016*

Categories	Total		HIV-infected		HIV-uninfected	
	Attributable fraction % (95% CI)	AF-adjusted prevalence %	Attributable fraction % (95% CI)	AF-adjusted prevalence %	Attributable fraction % (95% CI)	AF-adjusted prevalence %
Severe acute respiratory illness (SARI-7)						
Age (in years)†						
<1	92.9 (70.7–98.3)	3.9	92.3 (42.2–∞)‡	6.6	92.4 (68.3–98.2)	3.6
1–4	88.2 (75.0–94.5)	7.2	91.5 (67.4–97.8)	9.6	85.9 (66.1–94.1)	6.8
5–24	84.3 (66.6–92.6)	5.2	90.4 (60.7–97.1)	4.1	80.9 (49.4–92.7)	5.2
25–44	83.7 (58.1–93.7)	8.0	90.7 (58.1–97.6)	9.8	78.5 (19.3–95.9)	2.4
45–64	87.3 (63.3–95.6)	13.1	89.9 (55.0–97.7)	15.9	85.2 (29.1–96.9)	9.4
≥65	94.1 (54.6–99.2)	7.8	90.5 (48.2–∞)‡	8.2	90.8 (26.2–98.8)	7.4
<5	89.0 (79.1–94.2)	5.0	92.0 (70.4–97.8)	7.9	88.2 (75.3–94.3)	4.7
≥5	86.2 (77.4–91.6)	8.6	90.5 (79.0–95.7)	10.2	81.3 (63.8–90.3)	6.0
All	87.3 (81.3–91.4)	5.9	90.9 (82.0–95.4)	9.6	84.7 (75.3–90.6)	4.7
Influenza virus types/subtypes§						
A¶	86.7 (78.7–91.7)	4.1	91.3 (79.2–96.3)	5.8	81.9 (68.0–89.8)	3.4
A(H3N2)	93.6 (85.1–97.2)	2.9	94.6 (76.9–98.7)	4.2	92.1 (77.6–97.2)	2.4
A(H1N1)pdm09	80.7 (63.5–89.8)	1.3	90.9 (68.7–97.4)	1.8	71.7 (39.2–86.8)	1.1
B	88.4 (77.6–94.0)	1.9	91.8 (71.6–97.7)	3.9	89.0 (74.2–95.3)	1.2
Severe chronic respiratory illness (SCRI-7)						
Age (in years)†						
<1	95.7 (79.4–99.1)	7.4	92.9 (34.1–∞)‡	5.2	95.2 (75.4–99.1)	7.8
1–4	90.0 (69.2–96.8)	9.9	90.5 (48.2–99.2)	9.8	89.5 (62.6–97.1)	9.8
5–24	85.1 (65.1–93.7)	7.1	91.4 (40.1–98.7)	7.3	84.9 (54.1–95.1)	7.6
25–44	77.9 (41.8–91.6)	4.0	90.2 (33.4–96.1)	4.5	69.1 (18.3–92.9)	4.6
45–64	82.1 (48.9–93.7)	4.4	90.7 (9.3–95.1)	4.6	84.6 (31.7–96.5)	4.7
≥65	91.1 (29.3–98.9)	7.1	91.1 (17.3–∞)‡	9.4	88.1 (23.4–98.6)	6.3
<5	92.8 (82.9–96.9)	8.5	92.5 (50.3–98.9)	7.6	92.7 (80.6–97.3)	8.7
≥5	83.5 (71.9–90.3)	4.8	90.4 (64.5–93.1)	4.9	82.0 (63.6–91.1)	5.5
All	87.1 (79.4–91.9)	5.3	90.3 (69.4–93.5)	5.0	86.0 (76.8–92.7)	6.4
Influenza virus types/subtypes§						
A¶	82.7 (68.6–90.4)	3.1	83.3 (55.8–93.7)	3.0	81.1 (60.5–90.9)	3.2
A(H3N2)	93.1 (81.3–97.4)	2.0	94.8 (64.1–98.6)	1.9	92.7 (75.5–97.8)	2.1
A(H1N1)pdm09	64.4 (17.6–84.6)	1.0	74.2 (21.4–93.8)	1.2	52.8 (8.7–84.5)	0.8
B	91.5 (82.1–95.9)	2.1	92.4 (80.7–97.0)	1.8	88.9 (61.9–96.8)	3.0

*AF, attributable fraction.
†The overall influenza virus attributable fraction was obtained from models adjusted for any underlying medical conditions and the other respiratory viruses investigated in this study. Age and HIV infection were also included as predictors in the non-stratified models.
‡Estimated using exact logistic regression.
§The influenza virus attributable fraction by types/subtypes was obtained from models adjusted for age, HIV infection, any underlying medical conditions and the other respiratory viruses investigated in this study, including co-circulating influenza virus types and subtypes.
¶Includes influenza virus A not subtyped.

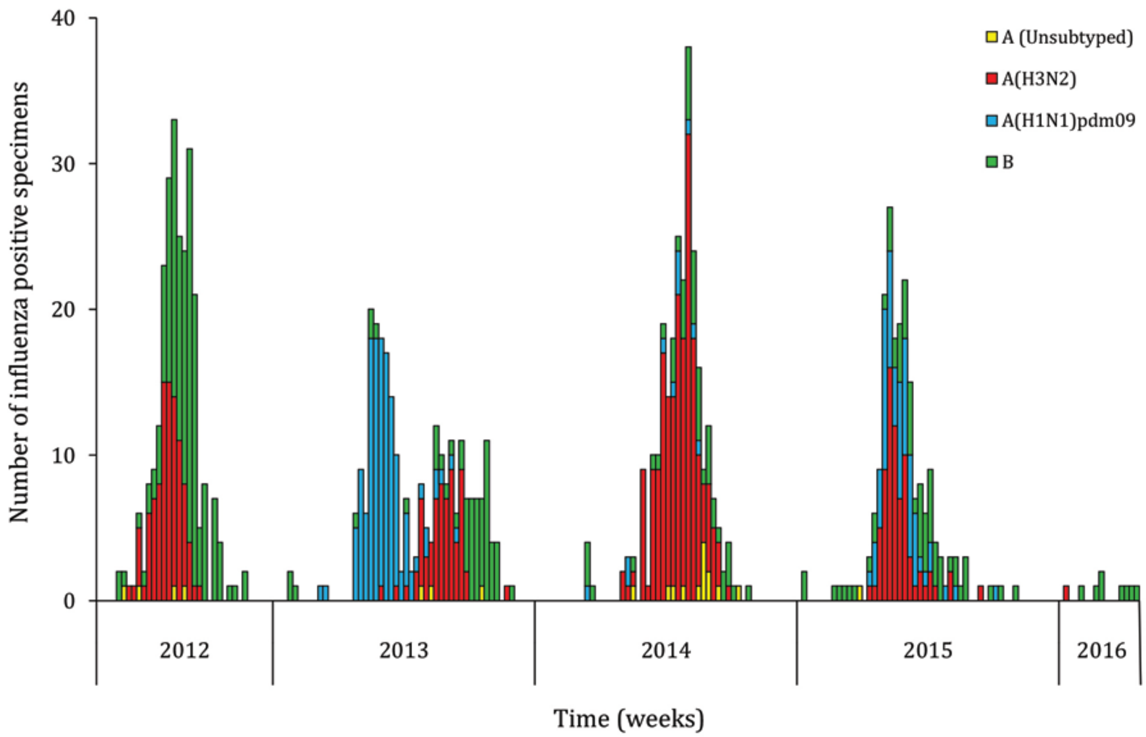

Technical Appendix Figure 1. Weekly number of influenza positive specimens by types and subtypes among asymptomatic individuals (controls), outpatients with influenza-like illness and inpatients with severe acute or chronic respiratory illnesses, Klerksdorp and Pietermaritzburg, South Africa, May 2012 – April 2016.

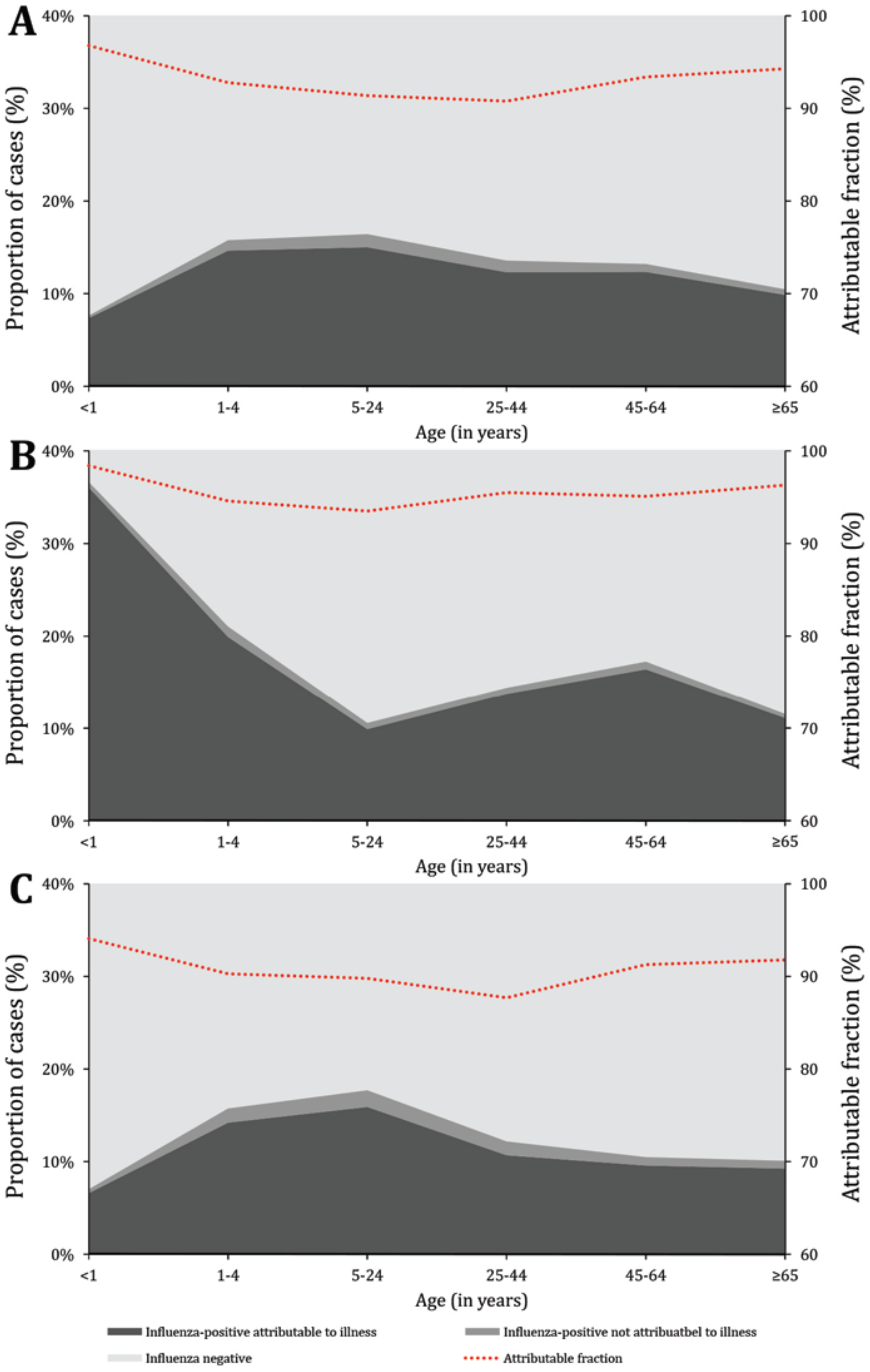

Technical Appendix Figure 2. Estimated influenza virus attributable fraction and proportion of influenza-positive cases attributable and not attributable to illness among outpatients with influenza-like illness, Klerksdorp and Pietermaritzburg, South Africa, May 2012 – April 2016. A: All patients; B: HIV-infected patients; and C: HIV-uninfected patients.

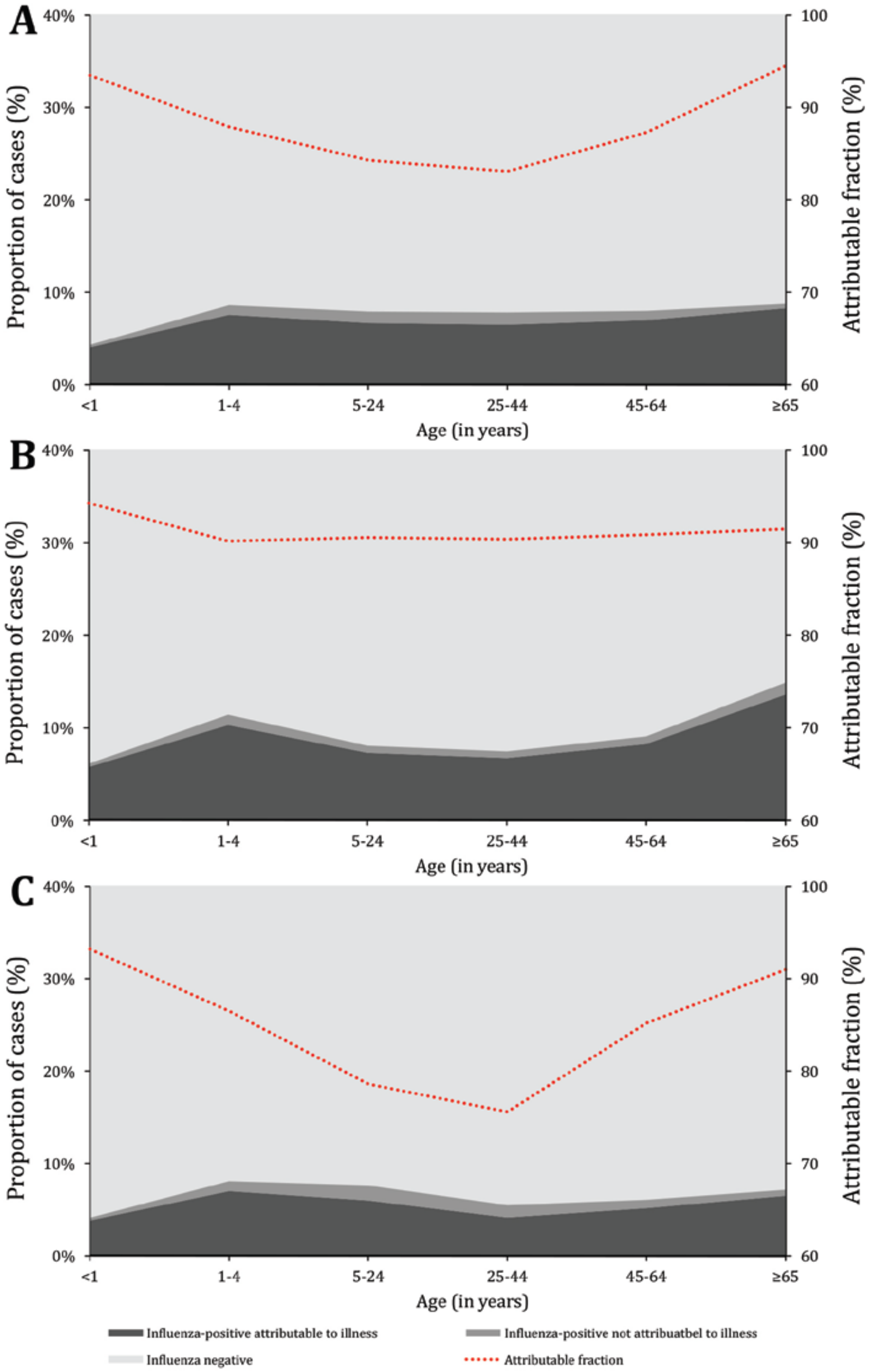

Technical Appendix Figure 3. Estimated influenza virus attributable fraction and proportion of influenza-positive cases attributable and not attributable to illness among inpatients with severe acute respiratory illness (symptoms duration ≤10 days), Klerksdorp and Pietermaritzburg, South Africa, May 2012 – April 2016. A: All patients; B: HIV-infected patients; and C: HIV-uninfected patients.

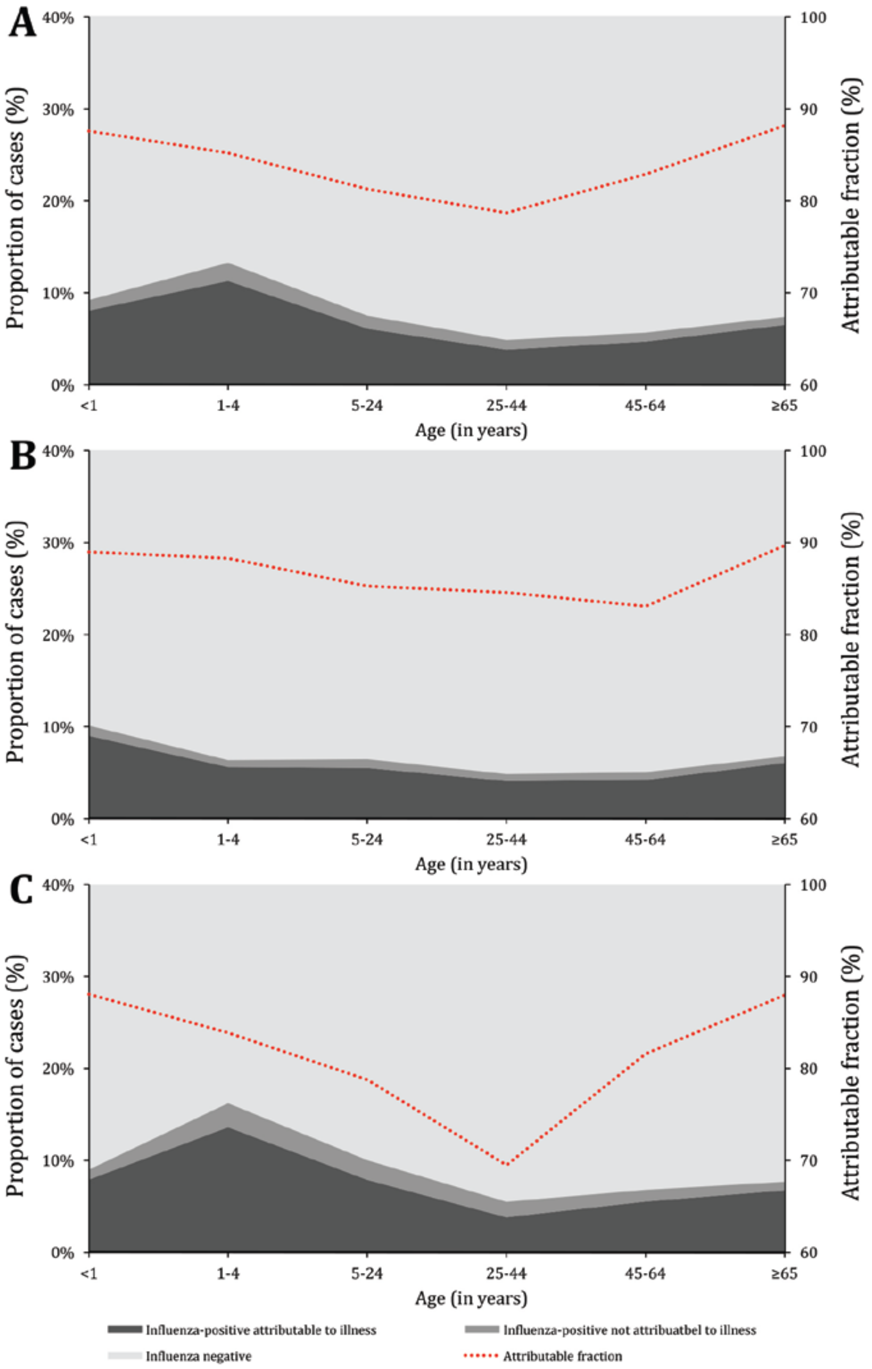

Technical Appendix Figure 4. Estimated influenza virus attributable fraction and proportion of influenza-positive cases attributable and not attributable to illness among inpatients with severe chronic respiratory illness (symptoms duration >10 days), Klerksdorp and Pietermaritzburg, South Africa, May 2012 – April 2016. A: All patients; B: HIV-infected patients; and C: HIV-uninfected patients.
